# The generalized inference on the ratio of mean differences for fraction retention noninferiority hypothesis

**DOI:** 10.1371/journal.pone.0234432

**Published:** 2020-06-09

**Authors:** Hsin-Neng Hsieh, Hung-Yi Lu

**Affiliations:** 1 Department of Statistics and Information Science, Fu Jen Catholic University, New Taipei City, Taiwan; 2 Office of Institutional Research, Fu Jen Catholic University, New Taipei City, Taiwan; Chang Gung Memorial Hospital at Linkou, TAIWAN

## Abstract

The fraction retention non-inferiority hypothesis is often measured for the ratio of the effects of a new treatment to those of the control in medical research. However, the fraction retention non-inferiority test that the new treatment maintains the efficacy of control can be affected by the nuisance parameters. Herein, a heuristic procedure for testing the fraction retention non-inferiority hypothesis is proposed based on the generalized *p*-value (GPV) under normality assumption and heteroskedasticity. Through the simulation study, it is demonstrated that, the performance of the GPV-based method not only adequately controls the type I error rate at the nominal level but also is uniformly more powerful than the ratio test, Rothmann’s and Wang’s tests, the comparable extant methods. Finally, we illustrate the proposed method by employing a real example.

## Introduction

The purpose of drug development in clinical trials is often to prove that the experimental treatment (new therapy) is superior to active control (standard therapy) or placebo. From an ethical point of view, for mortality or severe morbidity trials, as long as the effective treatment exist for conditions that can lead to death or severe irreversible morbidity, the placebo or untreated controls cannot be used. If ethically justifiable, it may be advisable to include a placebo group for internal validation. [1, 2]Traditionally, in clinical trials, the clinical medicine researchers hope to show that the experimental treatment is superior to the active control. However, when experimental treatments offer other advantages over controls (for example, better safety or ease of administration), then non-inferiority trial (NI trial) can be used for validation. [3] The goal of the NI trial is to conclude that the experimental treatment is more effective than a placebo and is not unacceptably less effective than the active control. The fixed margin method and the synthesis method are widely used to test non-inferiority hypothesis procedures in NI trials. The Food and Drug Administration (FDA) regulatory guidances have published the fixed margin and synthesis methods that are used in the design of NI trials. [4] The fixed margin method is to first define non-inferior margin, and then demonstrate that the experimental treatment is not worse than the control effect. [5] The purpose of the fraction retention hypothesis is to test what percentage of the active control effects the new treatment can retain. The synthesis method based on retention is used to test whether a new treatment can retain a fraction of the active control effects.

Some researchers have proposed methods for statistical verification of the retention NI hypothesis. For example, Rothmann *et al*. [6] proposed one test method, hereafter referred to as Rothmann’s test. Rothmann’s test assumes the standard drug’s efficacy to be positive, but in practice, this assumption is not necessarily reasonable. Moreover, the power of Rothmann’s test does not perform well on small sample size. Wang *et al*. [7] proposed another test that was developed from asymptotic normality theory, and hereafter, we refer to this test as Wang’s test. For the small sample size, Wang’s test is better than Rothmann’s test with respect to power. Additionally, in clinical trials, the sample size required for Wang’s test has been determined to be smaller than the size required for Rothmann’s test. Nevertheless, the proposed test statistic is assumed to have homogeneous variance under the alternative hypothesis as well as null hypothesis in Wang’s test. In practice, assuming homogeneity in NI trials is inappropriate. In recent years, Deng and Chen [8] derived a ratio test from the Cauchy-like distribution proposed by Masaglia [9]. In the ratio test, the standard drug efficacy is not assumed to be positive, and the test statistic is not assumed to have homogeneous variance under the alternative hypothesis as well as null hypothesis. In addition, the ratio test is more powerful than the two aforementioned tests. Nevertheless, in the ratio test, the critical value of the Cauchy-like distribution needs to be calculated by numerical integration, which makes the calculation process more complicated. Furthermore, the retention NI test procedure can be affected by the nuisance parameters. Because of the complexity of the sampling distribution of test statistics, it is difficult to assess the fraction retention non-inferiority hypothesis of ratio of mean differences. In the present study, a heuristic statistical testing procedure on the ratio of mean differences for the retention NI hypothesis is applied on the basis of the concept of GTVs. The heuristic statistical testing procedure is called the generalized *p* value based (GPV-based) method that is more convenient to calculate the type I error rate and empirical power without complicated computation than ratio test.

The *p*-value of a statistical test is calculated by using sample observations as the critical value for the test. If the nuisance parameters exist in the test procedure, the *p*-value may be dependent on the nuisance parameters and cannot be easily calculated. In order to overcome this problem, Tsui and Weerahandi [10] generalized the rejection region of the test so that the calculation of *p*-value can be independent of the nuisance parameters, and provided a GPV test procedure. Tsui and Weerahandi [10] gave the explicit definition of generalized test variable (GTV) and GPV, and showed that it is an exact probability of an extreme region. For the definition of GTV and GPV, please refer to the S1 Appendix.

Tsui and Weerahandi [10] successfully used the GPVs to provide small sample solution for hypothesis testing problems when nuisance parameters present and testing procedures are difficult to obtain. Subsequently, Weerahandi [11] provided the generalized pivotal quantities (GPQs) to construct the generalized confidence intervals (GCIs) of specific parameters which are contained the nuisance parameters. The GPVs has been successfully applied to various hypothesis testing topics, such as in research comparing accuracy by examining receiver operating characteristic curves in gold standard situations [12, 13], research on the tolerance interval for random effects models [14, 15], research on evaluation of dissolution profile similarity [16], research on GCIs in a linear measurement error model [17], research comparing accuracy by examining receiver operating characteristic curves when a gold standard situation does not exist [18], and research applying a delta-lognormal distribution to trawl survey data [19], and applying the generalized inference on the sign testing problem about the normal variance [20], etc.

In this study, the various methods on the ratio of mean differences for the fraction retention NI hypothesis are reviewed. We propose the GPV-based method which is a heuristic statistical testing procedure. Moreover, the type I error rate and empirical power of the GPV-based method are examined under simulation studies. We compare the GPV-based method’s performance with those from the Rothmann’s, Wang’s and the ratio tests. The methods are illustrated using published data. Conclusions are presented in final section. S1 and S2 Appendices detail the underlying definitions of generalized inference, and the reader is referred to [10] and [11].

## Ratio test, Rothmann’s test and Wang’s test

In this study, we use the same notation and definition as in Deng *et al*. [8] to compare the proposed method. In a three-arm NI trial, *T*_*NI*_, *C*_*NI*_ and *P*_*NI*_ represent the effects of experimental treatment, control, and placebo, respectively. The *C*_*H*_ and *P*_*H*_ respectively denote the effects of the control and placebo obtained from the historical trials. Let ${\hat{T}}_{NI}$, ${\hat{C}}_{NI}$, ${\hat{P}}_{NI}$, ${\hat{C}}_{H}$ and ${\hat{P}}_{H}$ represent the estimates of effects for *T*_*NI*_, *C*_*NI*_, *P*_*NI*_, *C*_*H*_ and *P*_*H*_, respectively. Define *θ*_1_ = *T*_*NI*_ − *P*_*NI*_ and *θ*_2_ = *C*_*H*_ − *P*_*H*_ to denote the effect of the new treatment and control, respectively. Hence, the estimates of *θ*_1_ and *θ*_2_ can be written as ${\hat{\theta }}_{1}={\hat{T}}_{NI}-{\hat{P}}_{NI}$ and ${\hat{\theta }}_{2}={\hat{C}}_{H}-{\hat{P}}_{H}$, respectively. In this research, we would like to analyze the fraction retention NI hypothesis testing problem in the following form,

$$\begin{array}{c} H_{0}:\delta \leq \delta_{0}\;\;\text{versus}\;\;H_{1}:\delta >\delta_{0}, \end{array}$$ (1)

where the null hypothesis is represented by *H*_0_, the alternative hypothesis is represented by *H*_1_, *δ* = *θ*_1_/*θ*_2_, and *δ*_0_(0 < *δ*_0_ < 1) denotes the given level of fraction retention. The hypothesis presented in (1) can be rewritten as

$$\begin{array}{c} H_{0}:1-(\frac{\theta_{2}-\theta_{1}}{\theta_{2}})\leq \delta_{0}\;\;\text{versus}\;\;H_{1}:1-(\frac{\theta_{2}-\theta_{1}}{\theta_{2}})>\delta_{0}. \end{array}$$ (2)

Next, we introduce the ratio, Rothmann’s and Wang’s tests.

Regarding the hypothesis presented in (1), Deng and Chen [8] proposed the ratio test on the basis of the Cauchy-like distribution proposed by Masaglia [9]. The ratio test is a non-standardized test, and for hypothesis (2), test statistic of the ratio test has the form

$$\begin{array}{c} \hat{Z}=\frac{{\hat{\theta }}_{1}}{{\hat{\theta }}_{2}}=1-\frac{\left({\hat{\theta }}_{2}-{\hat{\theta }}_{1}\right)}{{\hat{\theta }}_{2}}. \end{array}$$

Under the constancy assumption, namely *C*_*NI*_ − *P*_*NI*_ = *C*_*H*_ − *P*_*H*_, test statistic of the ratio test $\hat{Z}$ can be rewritten as

$$\begin{array}{c} \hat{Z}=1-\left({\hat{C}}_{NI}-{\hat{T}}_{NI}\right)/\left({\hat{C}}_{H}-{\hat{P}}_{H}\right). \end{array}$$ (3)

Furthermore, Deng and Chen [8] assumed that ${\hat{C}}_{NI}-{\hat{T}}_{NI}$ and ${\hat{C}}_{H}-{\hat{P}}_{H}$ are normally distributed with means *μ*_*NI*_ and *μ*_*H*_, and variances $\sigma_{NI}^{2}$ and $\sigma_{H}^{2}$. That is

$$\begin{array}{c} {\hat{C}}_{NI}-{\hat{T}}_{NI}∼N\left(\mu_{NI},\sigma_{NI}^{2}\right),{\hat{C}}_{H}-{\hat{P}}_{H}∼N\left(\mu_{H},\sigma_{H}^{2}\right), \end{array}$$

where *μ*_*NI*_ and *μ*_*H*_ indicate the mean effect of the new treatment relative to the active control in the NI trial and the mean effect of the active control relative to the placebo in the historical trial, respectively; $\sigma_{NI}^{2}$ and $\sigma_{H}^{2}$ indicate the variance of effect of the new treatment relative to the active control in the NI trial and the variance of effect of the active control relative to the placebo in the historical trial, respectively. And therefore,

$$\begin{array}{c} {\hat{T}}_{NI}-{\hat{P}}_{NI}=\left({\hat{C}}_{H}-{\hat{P}}_{H}\right)-\left({\hat{C}}_{NI}-{\hat{T}}_{NI}\right)∼N\left(\mu_{H}-\mu_{NI},\sigma_{H}^{2}+\sigma_{NI}^{2}\right). \end{array}$$

Let *θ*_1_ = *θ*_2_ − *μ*_*NI*_ and *θ*_2_ = *μ*_*H*_, the distribution of the estimated effect for new treatment relative to placebo can be indicated as

$$\begin{array}{c} {\hat{T}}_{NI}-{\hat{P}}_{NI}∼N\left(\theta_{1}=\theta_{2}-\mu_{NI},\sigma_{H}^{2}+\sigma_{NI}^{2}\right). \end{array}$$

Because of the complexity of the distribution of $\hat{Z}$ for the aforementioned NI hypothesis parameter settings, Deng and Chen [8] used the linear transformation method proposed by Marsaglia [9]. Accordingly, the distribution of $\hat{Z}$ is equivalence that of as follows:

$$\begin{array}{c} \hat{Z}∼r\cdot \left(a+X\right)/\left(b+Y\right)+1, \end{array}$$ (4)

where *a* = −*μ*_*NI*_/*σ*_*NI*_, *b* = *θ*_2_/*σ*_*H*_, and *r* = *σ*_*NI*_/*σ*_*H*_, *X* and *Y* are two independent standard normal random variables. In the NI trial, *a*, *b*, and *r* denote the standardized difference between the treatment and control effects, standardized efficacy of the control versus the placebo as determined in the historical data, and ratio of the *C*_*NI*_ − *T*_*NI*_ and *C*_*H*_ − *P*_*H*_ standard deviations, respectively.

Consider the parameter set (*b*, *r*, *δ*), let *g*_*b*,*r*, *δ*_(*t*) be the probability density function and *G*_*b*,*r*, *δ*_(*t*) be the cumulative distribution function of *r* ⋅ (*a* + *x*)/(*b* + *y*) + 1. Accordingly, $G_{b,r,\delta }^{-1}\left(t\right)$ denotes the quantile function of *G*_*b*,*r*, *δ*_(*t*). Under circumstances in which *b* and *r* are known, the ratio test has the following rejection region

$$\begin{array}{c} C_{Ratio\;test}=\{\hat{Z}>G_{b,r,\delta_{0}}^{-1}\left(1-\alpha \right)\}. \end{array}$$ (5)

If $\hat{Z}>G_{b,r,\delta_{0}}^{-1}\left(1-\alpha \right)$, the null hypothesis *H*_0_ displayed in (1) can be rejected. If *b* and *r* are unknown, the estimated values of *b* and *r* can be used to calculate the critical value of the rejection region for the ratio test.

Furthermore, for the fraction retention NI hypothesis presented in (1), Deng and Chen [8] derived the equivalence formulas of Rothmann’s test rejection region. The *C*_*Rothmann*′ *stest*_ denotes the rejection region of Rothmann’s test. The *C*_*Rothmann*′ *stest*_ is expressed as

$$\begin{array}{c} C_{Rothmann^{\prime }s\;test}=\{\frac{\left({\hat{C}}_{NI}-{\hat{T}}_{NI}\right)-\left(1-\delta_{0}\right)\left({\hat{C}}_{H}-{\hat{P}}_{H}\right)}{\sqrt{\sigma_{NI}^{2}+{\left(1-\delta_{0}\right)}^{2}\sigma_{H}^{2}}}<z_{\alpha }\}, \end{array}$$ (6)

where *z*_*α*_ is the upper *α* critical point of the standard normal distribution. One can reject *H*_0_ in (1) if $\frac{\left({\hat{C}}_{NI}-{\hat{T}}_{NI}\right)-\left(1-\delta_{0}\right)\left({\hat{C}}_{H}-{\hat{P}}_{H}\right)}{\sqrt{\sigma_{NI}^{2}+{\left(1-\delta_{0}\right)}^{2}\sigma_{H}^{2}}}<z_{\alpha }$.

In addition, for the fraction retention NI hypothesis stated in (1), Deng and Chen [8] derived the equivalence formulas of the rejection region of Wang’s test, which is denoted *C*_*Wang*′ *stest*_. The *C*_*Wang*′ *stest*_ can be expressed as

$$\begin{array}{c} C_{Wang^{\prime }s\;test}=\{\frac{(1-\frac{\left({\hat{C}}_{NI}-{\hat{T}}_{NI}\right)}{\left({\hat{C}}_{H}-{\hat{P}}_{H}\right)})-\delta_{0}}{\sqrt{\left(\sigma_{NI}^{2}+{\left(1-\delta_{0}\right)}^{2}\sigma_{H}^{2}\right)/{\left({\hat{C}}_{H}-{\hat{P}}_{H}\right)}^{2}}}>z_{1-\alpha }\}, \end{array}$$ (7)

where *z*_1−*α*_ is the upper 1 − *α* critical point of the standard normal distribution. The null hypothesis *H*_0_ displayed in (1) can be rejected if $\frac{(1-\frac{\left({\hat{C}}_{NI}-{\hat{T}}_{NI}\right)}{\left({\hat{C}}_{H}-{\hat{P}}_{H}\right)})-\delta_{0}}{\sqrt{\left(\sigma_{NI}^{2}+{\left(1-\delta_{0}\right)}^{2}\sigma_{H}^{2}\right)/{\left({\hat{C}}_{H}-{\hat{P}}_{H}\right)}^{2}}}>z_{1-\alpha }$.

In practice, the true values of $\sigma_{H}^{2}$, and $\sigma_{NI}^{2}$ are usually unknown, but corresponding estimates can be made on the basis of historical data and assumptions from current NI trials [8].

## Proposed method: GPV-based method

In this section, we proposed the heuristic statistical testing procedure on the ratio of mean differences for the retention NI hypothesis, which is based on the concept of GTVs. Let $X_{H}=\left\{X_{1},\ldots ,X_{n_{H}}\right\}$ and $Y_{NI}=\left\{Y_{1},\ldots ,Y_{n_{NI}}\right\}$ denote two independent random samples that have been drawn from normal distributions with means *μ*_*H*_, *μ*_*NI*_ and variances $\sigma_{H}^{2}$, and $\sigma_{NI}^{2}$, respectively. Without loss of generality, these random samples can be assumed to be mutually independent. We denote ${\bar{X}}_{H}$ and ${\bar{Y}}_{NI}$ as the sample means, $S_{H}^{2}$ and $S_{NI}^{2}$ as the sample variances. Moreover, ${\bar{x}}_{H}$, ${\bar{y}}_{NI}$, $s_{H}^{2}$ and $s_{NI}^{2}$ are the observed values of ${\bar{X}}_{H}$, ${\bar{Y}}_{NI}$, $S_{H}^{2}$ and $S_{NI}^{2}$, respectively.

According to (2), let *θ*_2_ = *μ*_*H*_ and *θ*_2_ − *θ*_1_ = *μ*_*NI*_, the hypothesis displayed in (2) can be rewritten as

$$\begin{array}{c} H_{0}:(\frac{\mu_{NI}}{\mu_{H}})\geq 1-\delta_{0}\;\;\text{versus}\;\;H_{1}:(\frac{\mu_{NI}}{\mu_{H}})<1-\delta_{0}. \end{array}$$ (8)

As result of the GTV can be constructed from the GPQ [11], to develop the GTV for the testing of the hypothesis presented in (8), we adopt Weerahandi’s concepts [11] to construct the GPQ for *μ*_*H*_ and *μ*_*NI*_. In regard to the definition of GPQ, please refer to the S2 Appendix. Hence, we define GPQs for *μ*_*H*_ and *μ*_*NI*_ as follows:

$$\begin{array}{cc} R_{\mu_{H}} & ={\bar{x}}_{H}-\frac{{\bar{X}}_{H}-\mu_{H}}{\sqrt{\frac{\sigma_{H}^{2}}{n_{H}}}}\sqrt{\frac{\sigma_{H}^{2}s_{H}^{2}}{n_{H}S_{H}^{2}}} \end{array}$$ (9)

$$\begin{array}{cc} & ={\bar{x}}_{H}-Z_{H}\sqrt{\frac{\left(n_{H}-1\right)s_{H}^{2}}{n_{H}U_{H}}}, \end{array}$$ (10)

$$\begin{array}{cc} R_{\mu_{NI}} & ={\bar{y}}_{NI}-\frac{{\bar{Y}}_{NI}-\mu_{NI}}{\sqrt{\frac{\sigma_{N}^{2}I}{n_{NI}}}}\sqrt{\frac{\sigma_{NI}^{2}s_{NI}^{2}}{n_{NI}S_{NI}^{2}}} \end{array}$$ (11)

$$\begin{array}{cc} & ={\bar{y}}_{NI}-Z_{NI}\sqrt{\frac{\left(n_{NI}-1\right)s_{NI}^{2}}{n_{NI}U_{NI}}}. \end{array}$$ (12)

where *Z*_*H*_ ∼ *N*(0, 1), *Z*_*NI*_ ∼ *N*(0, 1) and *U*_*H*_ ∼ *χ*^2^(*n*_*H*_ − 1), *U*_*NI*_ ∼ *χ*^2^(*n*_*NI*_ − 1). *χ*^2^(*n*_*H*_ − 1) and *χ*^2^(*n*_*NI*_ − 1) denote the chi-square distribution with degrees of freedom *n*_*H*_ − 1 and *n*_*NI*_ − 1, respectively. Also, *Z*_*H*_, *Z*_*NI*_, *U*_*H*_ and *U*_*NI*_ are mutually independent. From (10) and (12), $R_{\mu_{H}}$ and $R_{\mu_{NI}}$ have distributions that are free of parameters *μ*_*H*_, *μ*_*NI*_, $\sigma_{H}^{2}$, and $\sigma_{NI}^{2}$, respectively. Therefore, the Property D of the GPQ is fulfilled. When ${\bar{X}}_{H}$, ${\bar{Y}}_{NI}$, $S_{H}^{2}$ and $S_{NI}^{2}$ are substituted by their observed values ${\bar{x}}_{H}$, ${\bar{y}}_{NI}$, $s_{H}^{2}$ and $s_{NI}^{2}$ in (9) and (11), then $R_{\mu_{H}}$ and $R_{\mu_{NI}}$ turn out to be *μ*_*H*_ and *μ*_*NI*_. Therefore, $R_{\mu_{H}}$ and $R_{\mu_{NI}}$ fulfill the Property E of GPQ. Follow the above concepts, the GPQ for $\frac{\mu_{NI}}{\mu_{H}}$ is thus defined as

$$\begin{array}{cc} R_{\frac{\mu_{NI}}{\mu_{H}}} & =\frac{R_{\mu_{NI}}}{R_{\mu_{H}}}\\ & =\frac{{\bar{y}}_{NI}-\frac{{\bar{Y}}_{NI}-\mu_{NI}}{\sqrt{\frac{\sigma_{N}^{2}I}{n_{NI}}}}\sqrt{\frac{\sigma_{NI}^{2}s_{NI}^{2}}{n_{NI}S_{NI}^{2}}}}{{\bar{x}}_{H}-\frac{{\bar{X}}_{H}-\mu_{H}}{\sqrt{\frac{\sigma_{H}^{2}}{n_{H}}}}\sqrt{\frac{\sigma_{H}^{2}s_{H}^{2}}{n_{H}S_{H}^{2}}}} \end{array}$$ (13)

$$\begin{array}{cc} & =\frac{{\bar{y}}_{NI}-Z_{NI}\sqrt{\frac{\left(n_{NI}-1\right)s_{NI}^{2}}{n_{NI}U_{NI}}}}{{\bar{x}}_{H}-Z_{H}\sqrt{\frac{\left(n_{H}-1\right)s_{H}^{2}}{n_{H}U_{H}}}}. \end{array}$$ (14)

From (14), $R_{\frac{\mu_{NI}}{\mu_{H}}}$ has the distribution that is free of parameters. Also from (13), if the observable random variables ${\bar{X}}_{H}$, ${\bar{Y}}_{NI}$, $S_{H}^{2}$ and $S_{NI}^{2}$ are substituted by their observed values in $R_{\mu_{H}}$ and $R_{\mu_{NI}}$, respectively, then $R_{\frac{\mu_{NI}}{\mu_{H}}}$ becomes $\frac{\mu_{NI}}{\mu_{H}}$. Hence, $R_{\frac{\mu_{NI}}{\mu_{H}}}$ satisfies the requirements of being GPQ of $\frac{\mu_{NI}}{\mu_{H}}$.

Accordingly, we can construct a GTV for $\frac{\mu_{NI}}{\mu_{H}}$ given by

$$\begin{array}{cc} T_{\frac{\mu_{NI}}{\mu_{H}}} & =T(X_{1},\ldots ,X_{n_{H}},Y_{1},\ldots ,Y_{n_{NI}};x_{1},\ldots ,x_{n_{H}},y_{1},\ldots ,y_{n_{NI}};\frac{\mu_{NI}}{\mu_{H}})\\ & =R_{\frac{\mu_{NI}}{\mu_{H}}}-\frac{\mu_{NI}}{\mu_{H}}. \end{array}$$ (15)

For given data, the observed value of $R_{\frac{\mu_{NI}}{\mu_{H}}}$ is equal to $\frac{\mu_{NI}}{\mu_{H}}$, and $R_{\frac{\mu_{NI}}{\mu_{H}}}$ has the distribution that is free of parameters. Hence, the distribution of $T_{\frac{\mu_{NI}}{\mu_{H}}}$ does not depend on nuisance parameters for a specified value of $\frac{\mu_{NI}}{\mu_{H}}$, and the observed value of $T_{\frac{\mu_{NI}}{\mu_{H}}}$ is equal to zero. Therefore, Property A and Property B of a GTV are satisfied. Furthermore, the distribution function of $T_{\frac{\mu_{NI}}{\mu_{H}}}$ can be expressed as

$$\begin{array}{c} P(T_{\frac{\mu_{NI}}{\mu_{H}}}\leq t_{\frac{\mu_{NI}}{\mu_{H}}})=P(R_{\frac{\mu_{NI}}{\mu_{H}}}\leq 0) \end{array}$$ (16)

Because the probability function of $T_{\frac{\mu_{NI}}{\mu_{H}}}$ is stochastically increasing in $\frac{\mu_{NI}}{\mu_{H}}$, thus it fulfills Property C. Therefore, $T_{\frac{\mu_{NI}}{\mu_{H}}}$ is a GTV for $\frac{\mu_{NI}}{\mu_{H}}$. For the descriptions of Properties A, B and C, please refer to S1 Appendix.

In order to test $H_{0}:(\frac{\mu_{NI}}{\mu_{H}})\geq 1-\delta_{0}$ versus $H_{1}:(\frac{\mu_{NI}}{\mu_{H}})<1-\delta_{0}$, the required GPV is calculated using the following Monte Carlo algorithm:

**Step 1**: Select numerous Monte Carlo samples; for example, *M* = 10000. For 1 ≤ *m* ≤ *M*, generate mutually independent chi-square random variables *U*_*H*,*m*_ and *U*_*NI*,*m*_ with *n*_*H*_ − 1 and *n*_*NI*_ − 1 degrees of freedom, respectively. Additionally, generate mutually independent standard normal variables *Z*_*H*,*m*_ and *Z*_*NI*,*m*_, respectively.

**Step 2**: Calculate *R*_*μ*_*H*_, *m*_ and *R*_*μ*_*NI*_, *m*_ using (10) and (12).

**Step 3**: Calculate $R_{\frac{\mu_{NI}}{\mu_{H}},m}$ using (14).

**Step 4**: Finally, $T_{\frac{\mu_{NI}}{\mu_{H}},m}$ can be calculated from (15) for a specified 1 − *δ*_0_.

The GPV is thus estimated using the $p=\sum_{m=1}^{M}I\left(T_{\frac{\mu_{NI}}{\mu_{H}},m}\leq 1-\delta_{0}\right)/M$. For a fixed significance level *α*, if *p* < *α*, then $H_{0}:(\frac{\mu_{NI}}{\mu_{H}})\geq 1-\delta_{0}$ is rejected.

## Simulation studies

The simulation study includes three scenarios. First, the type I error rate obtained using the GPV-based method is compared with those obtained using the Ratio, Rothmann’s and Wang’s tests. Second, the empirical powers of the four tests are evaluated and the performances of the tests are compared. Third, the performances of the GPV-based method are compared under the different sample size.

### Simulation study I: Type I error rate

We make use of the same simulation parameters as in Deng and Chen [8] for comparison of GPV-based method, ratio test, Rothmann’s test and Wang’s test. The retention rate in the NI hypothesis is fixed at *δ*_0_ = 0.5 in this study. The mean effect of the active control relative to the placebo in the historical trial *μ*_*H*_ is set to be 0.24. To evaluate the type I error rate, the standardized control effect versus placebo in historical trial *b* is considered for three cases: (i) *b* = 2, (ii) *b* = 3, and (iii) *b* = 4. The *b*/*r* is represented as the mean efficacy of the active control relative to the placebo in historical trials divided by the standard deviation of the efficacy of the new treatment relative to the active control in the NI trial. The *b*/*r* is set to be 2, 4 and 8. Additionally, for the GPV-based method, the sample size is set to be 30.

Data for the simulation are independently generated 10000 times for each combination of parameters. The type I error rate is estimated for the data set generated by all methods. A total of 10000 Monte Carlo random sampling processes are performed for each data set for use in the GPV-based method. Given the 2.5% nominal significance level, the simulation study with 10000 random samples implies that the 97.5% of the type I error evaluated at either *δ*_0_ are between 0.0219 and 0.0281. Table 1 presents the type I error rates obtained from the simulations.

**Table 1:** Type I error rates obtained through simulation for Rothmann’s, Wang’s and Ratio tests as well as the GPV-based method.

*δ*_0_	*b*	*b*/*r*	Rothmann’s test	Wang’s test	Ratio test	GPV-based method
0.5	2	2	0.0235	0.0277	0.0239	0.0250
		4	0.0233	0.0307	0.0236	0.0242
		8	0.0240	0.0371	0.0232	0.0240
	3	2	0.0240	0.0240	0.0264	0.0248
		4	0.0231	0.0231	0.0244	0.0244
		8	0.0229	0.0235	0.0242	0.0242
	4	2	0.0240	0.0240	0.0265	0.0250
		4	0.0235	0.0235	0.0254	0.0248
		8	0.0233	0.0233	0.0254	0.0246
0.7	2	2	0.0242	0.0267	0.0244	0.0253
		4	0.0227	0.0272	0.0238	0.0250
		8	0.0226	0.0314	0.0236	0.0248
	3	2	0.0243	0.0243	0.0272	0.0246
		4	0.0242	0.0242	0.0262	0.0245
		8	0.0225	0.0226	0.0258	0.0242
	4	2	0.0244	0.0244	0.0261	0.0250
		4	0.0242	0.0242	0.0261	0.0245
		8	0.0227	0.0227	0.0257	0.0242
0.9	2	2	0.0252	0.0264	0.0263	0.0248
		4	0.0243	0.0262	0.0250	0.0245
		8	0.0242	0.0277	0.0239	0.0240
	3	2	0.0260	0.0260	0.0261	0.0248
		4	0.0249	0.0249	0.0267	0.0244
		8	0.0239	0.0239	0.0262	0.0242
	4	2	0.0262	0.0262	0.0262	0.0245
		4	0.0252	0.0252	0.0257	0.0244
		8	0.0243	0.0243	0.0260	0.0243
1.1	2	2	0.0273	0.0262	0.0261	0.0245
		4	0.0274	0.0258	0.0273	0.0240
		8	0.0273	0.0245	0.0276	0.0245
	3	2	0.0271	0.0269	0.0261	0.0250
		4	0.0274	0.0271	0.0256	0.0246
		8	0.0279	0.0275	0.0256	0.0243
	4	2	0.0269	0.0269	0.0259	0.0252
		4	0.0273	0.0273	0.0256	0.0245
		8	0.0274	0.0274	0.0252	0.0240
1.2	2	2	0.0274	0.0258	0.0273	0.0240
		4	0.0273	0.0245	0.0276	0.0245
		8	0.0261	0.0240	0.0276	0.0243
	3	2	0.0274	0.0271	0.0256	0.0246
		4	0.0279	0.0275	0.0256	0.0243
		8	0.0272	0.0265	0.0253	0.0244
	4	2	0.0273	0.0273	0.0256	0.0245
		4	0.0274	0.0274	0.0252	0.0240
		8	0.0273	0.0273	0.0254	0.0245

The type I error rates obtained using the GPV-based method are in the range (0.0240, 0.0253); the corresponding ranges obtained using the ratio, Rothmann’s and Wang’s tests are (0.0232, 0.0276), (0.0225, 0.0279) and (0.0226, 0.0371), respectively. Most of the type I error rates are suitably near the nominal level of 0.025. When *δ*_0_ < 1 and *δ*_0_ ≥ 1, the type I error rates obtained using the four tests are well controlled. However, for the GPV-based method, the percentage of frequencies with an type I error rate of less than 0.025 is higher than that for the other three tests. Consequently, regarding the statistical testing of the fraction retention NI hypothesis, the GPV-based method is capable of maintaining type I error rate close to the nominal level of 0.025 adequately.

### Simulation study II: Empirical power

**Fig 1:**
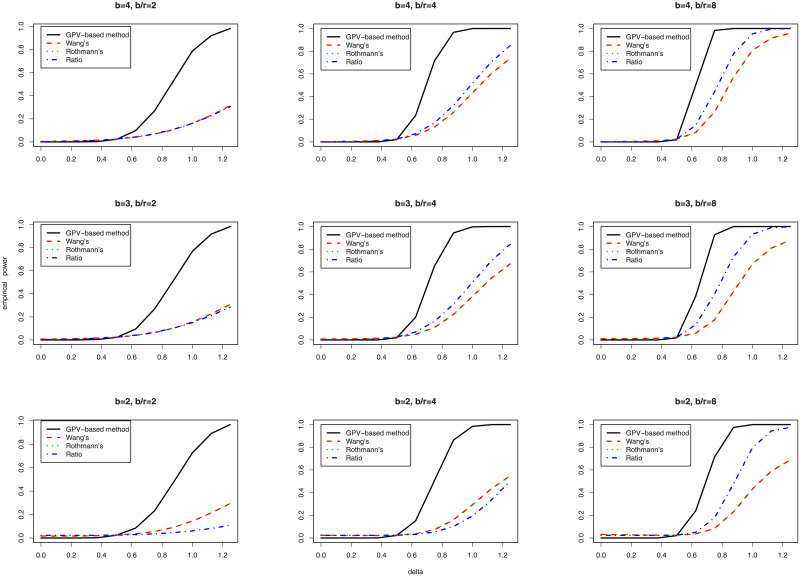
Empirical power functions of Rothmann’s test, Wang’s test and Ratio tests as well as the GPV-based method at *δ*_0_ = 0.5.

The GPV-based method’s empirical power is evaluated through another simulation study. We again employ the same simulation parameters as Deng and Chen [8]. The level of fraction retention *δ*_0_ is defined as 0.5. Fig 1 illustrates the results of the simulations.

When *b* and *b*/*r* are larger, the empirical power of the four methods is considerably greater. Simulation processes executed under different *b* and *b*/*r* values reveal that Rothmann’s test has similar empirical power to Wang’s test. Furthermore, the empirical power of the GPV-based method is uniformly more powerful than those of other three tests.

### Simulation study III: The performance of the GPV-based method under equal sample size

We conducted a simulation study of the performance by the GPV-based method under other sample sizes. We consider balanced sample sizes when *b* = 2, *b*/*r* = 2, 4, 8, *δ*_0_ = 0.5 and *δ*_1_ = 0.625. Table 2 details the type I error rate and empirical power simulation findings.

**Table 2:** The simulated Type I error rates and empirical powers of the GPV-based method under equal sample size, for *b* = 2, *b*/*r* = 2, 4, 8, *δ*_0_ = 0.5 and *δ*_1_ = 0.625.

	Type I Error Rate			Empirical Power		
*n*_*H*_ = *n*_*NI*_	*b*/*r* = 2	*b*/*r* = 4	*b*/*r* = 8	*b*/*r* = 2	*b*/*r* = 4	*b*/*r* = 8
30	0.0250	0.0242	0.0240	0.2151	0.4650	0.6568
40	0.0249	0.0245	0.0242	0.2768	0.5878	0.7928
50	0.0252	0.0248	0.0250	0.3431	0.6950	0.8786

In simulation study III, the GPV-based method is discovered to still exhibit sufficient size control at the nominal level under the different sample size. Furthermore, the empirical power increases with increasing sample size.

## Numerical example

In this section, we consider the Xeloda trial [21] presented by Rothmann *et al*. [4], Wang *et al*. [7], and Deng and Chen [8]. To make comparisons, we illustrate the analysis of the NI trial with the fraction retention NI hypothesis presented in (1).

Xeloda is an oral chemotherapeutic drug converted into 5-fluorouracil (5-FU). The approved first-line chemotherapy for metastatic colorectal cancer is 5-fluorouracil with leucovorin (5-FU/LV), which can be administered only through intravenous infusion. The Xeloda New Drug Application (NDA) was submitted to the Food and Drug Administration (FDA) in 2001 and contained two randomized trials. Each trial involved approximately 600 subjects and compared Xeloda (the new treatment) with 5-FU/LV (the control treatment). The NI hypothesis in these trials was that Xeloda would exert 50% or more of the effect of 5-FU/LV compared with 5-FU alone. Many studies on metastatic colorectal cancer have compared 5-FU/LV with 5-FU as the first-line treatment. In a random effects meta-analysis of 10 studies, the historical 5-FU/LV effect was estimated to be approximately 0.2341 and concomitant standard error to be approximately 0.0750 [21]. Hence, the observed standardized control effect was 3.1213. In a random-effects meta-analysis of 8 historical studies, the historical 5-FU/LV effect and corresponding standard error were estimated to be 0.2398 and 0.0593, respectively [21]. The observed standardized control effect was thus 4.0438.

Table 3 presents the analytic findings for two separate Xeloda trials and the pooled research obtained for the intent-to-treat (ITT) population, with calculations performed using Rothmann’s, Wang’s and Ratio tests, and GPV-based method. The *p*-values obtained for Rothmann’s, Wang’s and Ratio tests, and GPV-based method are displayed in Table 3. The results indicate that the *p*-values obtained for the GPV-based method are uniformly lower than those obtained for the other three tests. Therefore, the GPV-based method is uniformly more powerful than the other three tests in this case.

**Table 3:** Summary results for Xeloda noninferiority trial from Rothmann’s, Wang’s, and Ratio tests, and GPV-based method.

						*p*-value			
Historical Trials	Study (ITT)	*μ*_*NI*_	*σ*_*NI*_	$\hat{r}$	$\hat{\delta }$	Rothmann’s test	Wang’s test	Ratio test	GPV-based method
10 Control trials	S014695	-0.0036	0.0868	1.1573	1.0154	0.1010	0.1009	0.0830	0.0011
	S014796	-0.0844	0.0867	1.1560	1.3605	0.0165	0.0164	0.0140	0.0001
	Pooled	-0.0432	0.0613	0.8173	1.1845	0.0129	0.0128	0.0062	<0.0001
8 Control trials	S014695	-0.0036	0.0868	1.4637	1.0154	0.0891	0.0890	0.0774	0.0007
	S014796	-0.0844	0.0867	1.4621	1.3605	0.0129	0.0128	0.0110	0.0001
	Pooled	-0.0432	0.0613	1.0337	1.1845	0.0083	0.0083	0.0044	<0.0001

^†^ *μ*_*NI*_ = log*HR*(Xeloda/5-FU/LV), *σ*_*NI*_ = SE(log*HR*(Xeloda/5-FU/LV)).

## Conclusions and discussion

In this study, the GPV-based method is proposed for the construction of a procedure for testing the fraction retention NI hypothesis under normality assumption and heteroskedasticity. As a whole, through this study, under the different values of *b* and *b*/*r*, the results of empirical simulations reveal the GPV-based method to be able to exhibit adequate type I error rate control at the nominal level. The performance of the empirical power from the GPV-based method is also better than that of ratio, Rothmann’s and Wang’s tests. In addition, the GPV-based method is more concise in calculating the type I error rate and the empirical power than the ratio test. Therefore, based on the results of this study under normality assumption, the GPV-based method is suitable for recommendation to the problem about the fraction retention NI hypothesis test. Consequently, this is the reason why the concepts of GPV have been successfully applied in many situations including this study. A R program for computation of the GPV-based method is available from S3 Appendix.

Moreover, the GPV-based method is worth to apply under the normality assumptions, but may not be suitable for other probability models. The GPV-based method under other probability distribution assumptions needs further research in the future. However, one should note that under the normality assumption, the required percentiles of GPQ for $\frac{\mu_{NI}}{\mu_{H}}$ cannot be obtained in closed form, but can be estimated using Monte Carlo methods. Hence, the GPV-based method may less likely be used to estimate the optimal sample sizes.

## Supporting information

S1 Appendix Generalized test variables and generalized p-values. (PDF)

S2 Appendix Generalized pivotal quantities and generalized confidences. (PDF)

S3 Appendix The code of R program for computing the p-value by using GPV-based method. (PDF)
