# Supplementary material for: The generalized inference on the ratio of mean differences for fraction retention noninferiority hypothesis
Source: PLoS One. 2020 Jun 9;15(6):e0234432. doi: 10.1371/journal.pone.0234432 (PMC7282653; doi:10.1371/journal.pone.0234432)
Supplement: S1 Appendix — (PDF) [file pone.0234432.s001.pdf]

Supporting information

S1 Appendix. Generalized test variables and generalized p-values

This appendix details the basic concepts for generalized test variables and generalized p-values, which were developed by Tsui and Weerahandi [10].

$\mathbf{X}$ is a random variable that has the probability density function $f(\mathbf{X};\zeta)$, with $\zeta = (\theta, \eta)$, where θ constitutes our parameter of interest and η represents a vector that contains the nuisance parameters. Let $\mathbf{x}$ be the observed value of the random variable $\mathbf{X}$. If these three properties are fulfilled, then we can consider the following as a generalized test variable: $T = T(\mathbf{X}; \mathbf{x}, \zeta)$.

Property A: The distribution of T is free of nuisance parameters η.

Property B: The observed value of T, expressed as $t_{obs} = T(\mathbf{x}; \mathbf{x}, \zeta)$, is not depend on unknown parameters.

Property C: For fixed $\mathbf{x}$ and η, $P[T \leq t|\theta]$ is monotonic in θ for all t.

To test the null hypothesis $(H_0 : \theta \in \Theta_0)$ against the alternative hypothesis $(H_1 : \theta \in \Theta_1)$, we define the generalized extreme region $C = \{\mathbf{X} : T(\mathbf{X}; \mathbf{x}, \zeta) \geq t_{obs}\}$; therefore, there is low probability that C contains evidence rejecting the null hypothesis. At this stage, Θ_0 is a subset of the parameter space, with Θ_1 being its complement. Thus, a generalized p-value (GPV) is expressed as

$$p = \sup_{\theta \in \Theta_0} P[C|\theta].$$

Without loss of generality, we can test $H_0 : \theta \leq \theta_0$ against $H_1 : \theta > \theta_0$. If T is deemed increase stochastically in θ, we can conveniently write the following expression for the GPV:

$$p = P[T \geq t_{obs}|\theta_0].$$

In a test for the nominal level α, if $p < \alpha$, we have strong evidence for rejection of the null hypothesis. The actual T distribution function is generally too complex to obtain; therefore, Monte Carlo simulation is often employed to calculate the GPV.
