# Supplementary material for: The generalized inference on the ratio of mean differences for fraction retention noninferiority hypothesis
Source: PLoS One. 2020 Jun 9;15(6):e0234432. doi: 10.1371/journal.pone.0234432 (PMC7282653; doi:10.1371/journal.pone.0234432)
Supplement: S2 Appendix — (PDF) [file pone.0234432.s002.pdf]

Supporting information

S2 Appendix. Generalized pivotal quantities and generalized confidences

This appendix details the basic concepts of generalized pivotal quantities and generalized confidence intervals proposed by Weerahandi [11].

A random quantity $R = R(\mathbf{X}; \mathbf{x}, \zeta)$ is called a generalized pivotal quantity (GPQ) if following two properties hold.

Property D: The distribution of R is free of unknown parameters.

Property E: The observed value of R, $r_{obs} = R(\mathbf{x}; \mathbf{x}, \zeta)$, does not depend on nuisance parameters η.

Accordingly, the two-sided equal tailed $100(1-\alpha)\%$ generalized confidence interval for θ is given by $(R_{\alpha/2}, R_{1-\alpha/2})$, where $R_{\alpha/2}$ and $R_{1-\alpha/2}$ are the $100(\alpha/2)$th and $100(1-\alpha/2)$th percentiles of the distribution of R. Moreover, the percentiles of R can be estimated by using Monte Carlo algorithm.
