# Supplementary material for: The generalized inference on the ratio of mean differences for fraction retention noninferiority hypothesis
Source: PLoS One. 2020 Jun 9;15(6):e0234432. doi: 10.1371/journal.pone.0234432 (PMC7282653; doi:10.1371/journal.pone.0234432)
Supplement: S3 Appendix — (PDF) [file pone.0234432.s003.pdf]

Supporting information

S3 Appendix. The code of R program for computing the *p*-value by using GPV-based method

```
b=2
br=2
r=b/br
delta0=0.5
delta1=0.5
muh=0.24
sigmah=0.24/b
muni=muh-delta1*muh
sigmani=0.24*r/b
n.ni=30
n.h=30

n=10000
G=matrix(NA,n,1)
for(i in 1:n)
{
CHIH=rchisq(n,n.h-1)
CHINI=rchisq(n,n.ni-1)
Z1=rnorm(n,0,1)
Z2=rnorm(n,0,1)
NI=rnorm(n.ni,muni,sigmani)
H=rnorm(n.h,muh,sigmah)
R21=mean(NI)-Z1*sqrt(((n.ni-1)*var(NI))/(n.ni*CHINI))
R2=mean(H)-Z2*sqrt(((n.h-1)*var(H))/(n.h*CHIH))
GPQ=R21/R2
G[i,]=quantile(GPQ,0.975)
}
A=ifelse(G<(1-delta0),1,0)
g.pvlue=sum(A)/n
g.pvalue
```
